# Approaches for Recognizing Disease Genes Based on Network

**DOI:** 10.1155/2014/416323

**Published:** 2014-02-24

**Authors:** Quan Zou, Jinjin Li, Chunyu Wang, Xiangxiang Zeng

**Affiliations:** ^1^School of Information Science and Technology, Xiamen University, Xiamen 361005, China; ^2^School of Computer Science and Technology, Harbin Institute of Technology, Harbin 150001, China

## Abstract

Diseases are closely related to genes, thus indicating that genetic abnormalities may lead to certain diseases. The recognition of disease genes has long been a goal in biology, which may contribute to the improvement of health care and understanding gene functions, pathways, and interactions. However, few large-scale gene-gene association datasets, disease-disease association datasets, and gene-disease association datasets are available. A number of machine learning methods have been used to recognize disease genes based on networks. This paper states the relationship between disease and gene, summarizes the approaches used to recognize disease genes based on network, analyzes the core problems and challenges of the methods, and outlooks future research direction.

## 1. Introduction

Although the human genome project has been accomplished and has achieved great success, and new methods that verify gene function with high-throughput have been applied, studying genetic problems that induce diseases is still one of the major challenges facing humanity [1]. The traditional gene mapping method is based on family genetic disease. First, genes inducing diseases are located in a chain interval. Most recent studies at recognizing disease gene that involves linkage analysis or association studies have resulted in a genomic interval of 0.5 cm to 10 cm, which contains 300 genes [2, 3]. Second, using the biological experiment method to identify each gene located in a chain interval requires a large number of human resources and capital support [4]. In addition, recognizing disease gene by checking the genes set in the interval is often not possible [5]. However, the study of candidate association works well when using a set of known functional candidate genes, which have a clear biological relationship to the disease [6]. Selecting known functional candidate genes is not easy and is often limited by a good deal of factors. The selection of functional candidate genes and prioritization candidate genes has been one of the keys in recognizing disease genes because several reorganization approaches are based on the functions of these genes.

In recent years, a number of recognizing disease gene approaches and computer tools have been developed through building mathematic models based on functional annotation, sequence-based features, protein interaction, and disease phenotype [7–16], such as sequence features [15], functional annotation [7, 8, 10, 13], and physical interactions [12, 13, 17]. Based on the above features, an approach to rank candidate disease genes by computing a correlation score that stands for the correlation between genes and diseases has been introduced. However, various factors may affect the association between genes and diseases.

System biology has indicated that diseases with overlapping clinical manifestations are induced by one or more mutations from the same function module [18–21]. Researches in biological experiments of human disease and patterns have found that genes causing similar disease phenotype often interact with each other directly or indirectly [22–24]. These discoveries have shown that positive correlation exists between disease phenotype and disease gene. Many researchers have proposed disease gene prediction methods based on gene interaction and disease phenotype similarity [7, 25–29]. Recently, many approaches making full use of gene interaction and disease similarity have established the gene interaction and disease phenotype similarity network to predict disease genes. Some typical methods based on networks will be introduced in detail in this paper.

## 2. Datasets

In the field of biological information, construction dataset is the data foundation of all subsequent work. The validity of datasets directly affects the validity and reliability of the learning algorithm and test. Thus, building a dataset is a basic and important preparatory work.

The recognition of disease gene datasets is mainly obtained from two databases: Online Mendelian Inheritance in Man (OMIM), which is a synthesis database [30–32], and Human Protein Reference Database (HPRD) [33, 34]. Although none of the datasets from OMIM or HPRD are currently complete, they are comprehensive enough [6].

OMIM has the most abundant information, most extensive resources, most comprehensive, authoritative, and timely human genes and genetic disorders based on knowledge composed to support human genetics research and education and the clinical genetics research. OMIM is daily updated and has free access and acquisition at http://www.omim.org/. In OMIM, each item has a short text summary of a generally determined phenotype or gene and a large number of links to other genetic databases [30]. Datasets of disease phenotype and gene-disease phenotype can be obtained from OMIM. However, the data from OMIM need to be disposed to recognize the disease genes [35].

HPRD is a database which curetted proteomic information suited to human proteins. Even though HPRD is updated relatively slow, it is a full-scale resource for studying the relationship between human diseases and genes [36] and is linked to an outline of human signaling paths. HPRD is also available for free at http://www.hprd.org/ [34]. The dataset of gene interaction can be obtained from HPRD [35].

## 3. Networks

Most research on recognizing disease genes use networks, including the disease phenotype network, protein-protein interaction network, and gene-disease phenotype network, among others. In this study, we introduce only the most commonly used networks. *G*_*PPI*_ represents the gene (proteins) interaction network, *G*_*DP*_ represents the disease phenotype network, and *G*_*P*-*DP*_ represents the gene-disease phenotype network [6, 16].

**Figure 1:**
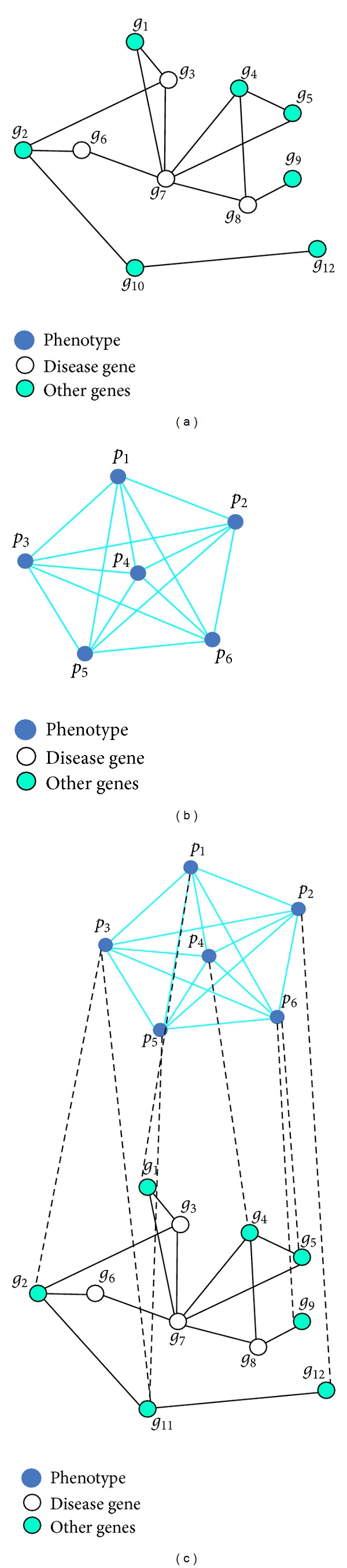
Illustration of the network by using a specific example: (a) is a gene-gene network, (b) is a disease phenotype network, and (c) is a gene-disease phenotype network [6].

1. *G*_*PPI*_ = (*G*, *E*_*G*_) is an undirected graph and denotes the gene-gene interaction. *G* = {*g*_1_, *g*_2_,…, *g*_*n*_} is the subset of the gene set, and *E*_*G*_ ⊂ *G* × *G* expresses the interaction of genes with weight.  Figure 1(a) shows *G*_*PPI*_. In the gene-gene interaction network, the relationship between genes is obtained from the gene-gene relationship database, which is one of the most important databases in the biological information field.
2. *G*_*DP*_ = (*D*, *E*_*D*_) is also an undirected graph and denotes the disease phenotype network. *D* = {*d*_1_, *d*_2_,…, *d*_*n*_} is the subset of the disease phenotype set, and *E*_*D*_ ⊂ *D* × *D* represents the similarity of the disease phenotype with weight.  Figure 1(b) shows *G*_*DP*_. In the disease phenotype network, the relationship between disease phenotypes is obtained from the phenotype relationship database, which can also be replaced by the disease-disease relationship database.
3. *G*_*P*-*DP*_ = (*G*, *D*, *E*_*P*-*DP*_), which is an undirected biograph, is a gene-disease phenotype network. *G* is the subset of the gene set, and *D* is the subset of the disease phenotype set. *E*_*P*-*DP*_ ⊂ *G* × *D* expresses the link between the known gene and the disease phenotype.  Figure 1(c) stands for *G*_*P*-*DP*_. The association between disease gene and disease phenotype can be obtained from the gene-disease relationship database.

## 4. Methods

In previous research, various methods, such as CIPHER, RWRH, Prince, Meta-path, Katz, Catapult, Diffusion Kernel [5], and ProDiGe, were used to recognize disease genes. In the current paper, we introduce several types of typical recognition disease gene methods.

### 4.1. CIPHER

CIPHER [6] is a tool for predicting and prioritizing disease genes. Furthermore, CIPHER is applied to general genetic phenotypes, which do better in the genome-wide scan of disease genes; furthermore, they are extendable for exploring gene cooperatives in complex diseases. CIPHER is based on an assumption that if two genes have the closest connection in the gene interaction, then the two genes can lead to more similar phenotypes. A regression model can be formulated according to this assumption. A score assessing how likely a gene is associated with a specific phenotype is obtained from the regression model. To construct the regression model, the similarity between phenotypes, interaction between proteins and genes, and list of associations between known disease gene and phenotype must be prepared. The next paragraph expresses the procedures of prioritizing disease genes.

For a given query phenotype and candidate genes, CIPHER first combines the gene interaction network, disease phenotype network, and gene-disease phenotype network into a single network. The similarity scores of the query phenotype with all known phenotypes in the disease phenotype network are derived directly from the phenotype network and the topological distances between the candidate genes. All known disease genes in the gene interaction network are counted and grouped on the basis of their phenotypes. The correlation between phenotypes and disease genes is obtained and acts as the concordance score for each candidate gene by using the regression model. Finally, all candidate genes for the query phenotype are ranked in line with the concordance scores. On account of different neighborhood systems, two ways are available to define the topological distance: direct neighbor and shortest path. Thus, there are two versions of CIPHER which are CIPHER-SP and CIPHER-DN [6].

The similarity scores of the query phenotype and all phenotypes in the disease phenotype network are calculated by the following formulation:

(1) $$\begin{array}{c} S_{{pp}^{\prime }}=C_{p}+\sum_{g\in G(p)}\sum_{g^{\prime }\in G(p^{\prime })}\beta_{pg}e^{-L_{gg^{\prime }}^{2}}. \end{array}$$

In the above formulation, *S*_*pp*′_ is the similarity score of the query phenotype and another phenotype in the disease phenotype network. *L*_*gg*′_ is the topological distance between the candidate genes and *g*′ in the gene interaction network. There exit two ways to define the topological distance, *L*_*gg*′_, on the basis of how to consider indirect the interaction. One way to define the topological distance is shortest path; *L*_*gg*′_ is the graph theory shortest path length between genes *g* and *g*′ in the gene interaction network. The other way to define the topological distance is direct neighbor; *L*_*gg*′_ is infinity when *g* and *g*′ are indirect neighbors. *G*(*p*) indicates all disease genes belonging to phenotype *p*. *C*_*p*_ is a constant and can act as the basal similarity between *p* and other phenotypes whose causative genes are not connected to those of *p* in the gene interaction network. *β*_*pg*_ is the coefficient of the regression model and stands for the level of gene *g* contributing to the similarity of the phenotype *p* to any other phenotype *p*′. To denote the association between a phenotype and a gene, the following formulation (2) is defined:

(2) $$\begin{array}{c} \Phi_{gp^{\prime }}=\sum_{g^{\prime }\in G(p^{\prime })}e^{-L_{gg^{\prime }}^{2}}. \end{array}$$

The following vector is used to denote the similarities between the query phenotype and all phenotypes in the disease phenotype network: *S*_*p*_ = (*S*_*pp*_1__, *S*_*pp*_2__, *S*_*pp*_3__,…, *S*_*pp*_*n*__).

In the same way, the following vector is used to denote the closeness between the genes and the phenotypes in the disease phenotype network: Φ_*g*_ = (Φ_*gp*_1__, Φ_*gp*_2__, Φ_*gp*_3__,…, Φ_*gp*_*n*__). Synthesizing Formulas (1) and (2) and two vectors extends Formula (1) to the following form:

(3) $$\begin{array}{c} S_{p}=C_{p}+\sum_{g\in G(p)}\beta_{pg}\Phi_{g}. \end{array}$$

In this regression model, the concordance score is defined by Formula (4):

(4) $$\begin{array}{c} CS_{pg}=\frac{\mathrm{cov}\left(S_{p},\Phi_{g}\right)}{\sigma \left(S_{p}\right)\sigma \left(\Phi_{g}\right)}, \end{array}$$

where cov⁡ and *σ* are the covariance and standard deviation, respectively. The candidate genes for the query phenotype are ranked according to the values obtained from Formula (4). If a gene that does not connect to any disease genes exists, then Formula (4) cannot be used and the gene will rank at the tail.

### 4.2. PRINCE

PRINCE is another approach based on networks for ranking candidate disease genes for a given disease and inferring the complex associations between genes. PRINCE is on account of formulating constraints on the ranking function that involved usage of prior information and its smoothness over the network.

Before using PRINCE, the gene disease composed of the phenotype network (set of gene-disease associations), gene-gene interaction network (set of gene-gene association), and at least a query disease phenotype is prepared. *G* = (*V*, *E*, *w*) denotes the gene-gene interaction network, where *V* is the set of genes, *E* is the set of interactions, and *w* is a weight function denoting the reliability of each interaction. Given a query disease phenotype, PRINCE ranks all the genes in *V*.

Suppose a gene *v* ∈ *V*, the direct neighborhood of gene *v* is denoted by *N*(*v*). The prioritization candidate disease gene function is denoted by *F* : *V* → *ℜ*, and *F*(*v*) = *q* reflects the relevance of *v* to *q*. Another function is defined as prior knowledge function denoted by *Y* : *V* → {0,1}. In the prior knowledge function, gene *v* is related to *q*, *V*(*v*) = 1; otherwise, *V*(*v*) = 0. PRINCE computes function *F* that is smooth over the network. Thus, function *F* is a combination of two conditions:

(5) $$\begin{array}{c} F\left(v\right)=\alpha \left[\sum_{u\in N(v)}F\left(u\right)w^{\prime }\left(v,u\right)\right]+\left(1-\alpha \right)Y\left(v\right), \end{array}$$

where the parameter *α* ∈ (0,1) weighs the relative importance of gene *v* to gene *u*. *w*′ is a normalized form of *w*. Formally, a diagonal matrix *D* is defined, and *D*(*i*, *i*) is the sum of row *i* of *W*. *W* is normalized by *W*′ = *D* ^−1/2^ *WD* ^−1/2^, which obtains a symmetric matrix. Here, $W_{ij}^{\prime }=W_{ij}/\sqrt{D\left(i,i\right)D\left(j,j\right)}$. Formula (5) can be expressed in linear form as follows:

(6) $$\begin{array}{c} F=\alpha W^{\prime }F+\left(1-\alpha \right)Y⟺F={\left(I-\alpha W^{\prime }\right)}^{-1}\left(1-\alpha \right)Y, \end{array}$$

where *F* and *V* are viewed as vectors of size |*V*|. *W*′ is a matrix whose values are given by *w*′. Given that the eigenvalues of *W*′ are set in [−1, 1], *α* ∈ (0,1), and the eigenvalues of (*I* − *αW*′) are positive. In addition, (*I*−*αW*′)^−1^ exists.

The above linear system can be solved accurately because an iterative propagation-based algorithm works fast and is guaranteed to converge to the system solution for larger networks. Formula (6) is transferred to an iterative algorithm and is denoted as follows:

(7) $$\begin{array}{c} F^{t}=\alpha W^{\prime }F^{t-1}+\left(1-\alpha \right)Y, \end{array}$$

where *F* ^1^ = *Y*. Every node propagates the information received in the previous iteration to its neighbors. Finally, the values obtained from Formula (7) rank all the candidate disease genes for a query disease phenotype.

### 4.3. RWRH

Random walk with restart on heterogeneous network (RWRH) is extended from the random walk with restart algorithm to the heterogeneous network. The heterogeneous network is constructed by connecting the gene-gene interaction network and disease phenotype network by using the gene-disease phenotype relationship information. In brief, the gene-disease phenotype network is the heterogeneous network. RWRH prioritizes the genes and the phenotypes simultaneously, which is inspired by the coranking framework [37]. Given a query disease, seed nodes as genes and phenotypes are associated with the disease, and the top ranked phenotype is the most similar to the query disease.

Random walk is defined as an iterative walker transition from its current node to a randomly selected neighbor, starting at a given source node *v* in the network. However, RWRH allows the restart of the walk in every time step at node *v* with probability *r*. *P*_0_ is the probability vector at step  0, indicating that it is the initial probability vector with the sum of probabilities equal to 1. Similarly, *P*_*s*_ is the probability vector at step *s*, in which the *i*th element holds the probability of finding the random walker at node *i* at step *s*. The probability vector at step *s* + 1 is denoted as follows:

(8) $$\begin{array}{c} P_{s+1}=\left(1-\gamma \right)M^{T}P_{s}+\gamma P_{0}, \end{array}$$

where *M* is the transition matrix of the heterogeneous network; *M*_*ij*_ is the transition probability from node *i* to node *j*; *γ* ∈ (0,1) is the restart probability in every time step. After several iterations, *P*_*∞*_ reaches a steady-state that is obtained by performing the iteration until the change between *P*_*s*_ and *P*_*s*+1_ falls below 10^−10^. *P*_*∞*_ is the measure of closing to seed nodes. In vector *P*_*∞*_, when *P*_*∞*_(*i*) > *P*_*∞*_(*j*), node *i* is more likely to be the seed node than node *j*.

*M* is the transition matrix of the heterogeneous network. In addition, *M* consists of four subnetwork transition networks and is denoted as follows:

(9) $$\begin{array}{c} M=\left[\begin{array}{cc} M_{G} & M_{GP}\\ M_{PG} & M_{p} \end{array}\right], \end{array}$$

where *M*_*G*_ is the transition matrix of the gene-gene interaction network, which is the intrasubnetwork of the heterogeneous network. *M*_*p*_ is the transition matrix of the disease phenotype network, which is also the intrasubnetwork of the heterogeneous network. *M*_*PG*_ and *M*_*GP*_ are the intersubnetwork transition matrixes. Supposing the probability of jumping from gene-gene interaction network to the disease phenotype network is *λ*, the reverse is the same. In the gene-gene interaction network, *λ* = 0 if a node is not connected to the phenotype. If a node is directly linked to the disease phenotype network, then the node will jump to the disease phenotype network with probability *λ*. The node will jump to other nodes in the gene-gene interaction network with probability 1 − *λ*. Thus, the transition probability from *g*_*i*_ to *p*_*j*_ can be denoted as follows:

(10) $$\begin{array}{c} {\left(M_{GP}\right)}_{i,j}=P\left(p_{j}\;|\;g_{i}\right)=\{\begin{array}{cc} \frac{\lambda B_{ij}}{\sum_{j}B_{ij}}, & \text{if}\;\underset{j}{\sum }B_{ij}\neq 0\\ 0, & \text{otherwise.} \end{array} \end{array}$$

In the same way, the transition probability from *p*_*i*_ to *g*_*j*_ can be denoted as follows:

(11) $$\begin{array}{c} {\left(M_{PG}\right)}_{i,j}=P\left(g_{j}\;|\;p_{i}\right)=\{\begin{array}{cc} \frac{\lambda B_{ji}}{\sum_{j}B_{ji}}, & \text{if}\;\underset{j}{\sum }B_{ji}\neq 0\\ 0, & \text{otherwise.} \end{array} \end{array}$$

The transition probability from *g*_*i*_ to *g*_*j*_, which is the element of *M*_*G*_ at the *i*th row and *j*th column, can be denoted as follows:

(12) $$\begin{array}{c} {\left(M_{G}\right)}_{i,j}=\{\begin{array}{cc} \frac{{\left(A_{G}\right)}_{i,j}}{\sum_{j}{\left(A_{G}\right)}_{i,j}}, & \text{if}\;\underset{j}{\sum }B_{ij}=0\\ \frac{\left(1-\lambda \right){\left(A_{G}\right)}_{i,j}}{{\sum_{j}\left(A_{G}\right)}_{i,j}}, & \text{otherwise.} \end{array} \end{array}$$

The transition probability from *p*_*i*_ to *p*_*j*_, which is the element of *M*_*P*_ at the *i*th row and the *j*th column, can be denoted as follows:

(13) $$\begin{array}{c} {\left(M_{P}\right)}_{i,j}=\{\begin{array}{cc} \frac{{\left(A_{P}\right)}_{i,j}}{\sum_{j}{\left(A_{P}\right)}_{i,j}}, & \text{if}\;\underset{j}{\sum }B_{ij}=0\\ \frac{\left(1-\lambda \right){\left(A_{P}\right)}_{i,j}}{{\sum_{j}\left(A_{P}\right)}_{i,j}}, & \text{otherwise.} \end{array} \end{array}$$

In the above four formulations, *A*_*G*(*n*×*n*)_, *A*_*P*(*m*×*m*)_, and *B*_(*n*×*m*)_ are the adjacency matrixes for the gene-gene interaction network, disease phenotype network, and gene-disease phenotype network, respectively. The adjacency matrix of the heterogeneous network can be denoted as follows:

(14) $$\begin{array}{c} A=\left[\begin{array}{cc} A_{G} & B\\ B^{T} & A_{P} \end{array}\right]. \end{array}$$

The initial probability of the gene-gene interaction network and phenotype network is denoted by *u*_0_ and *v*_0_, respectively. The initial probability of the gene network *u*_0_ makes the equal probabilities to all the seed nodes in the gene network, with the sum of the probabilities equal to 1. The initial probability of the phenotype network *v*_0_ is the same as the gene-gene interaction network. Thus, the initial probability vector of the heterogeneous network is denoted as *P*_0_ = [(1−*η*)*u*_*∞*_ *ηv*_*∞*_]^*T*^. In the initial probability vector of the heterogeneous network, the parameter *η* ∈ (0,1) acts as the judge to weight the importance of each subnetwork. When *η* = 0.5, the importance of the gene-gene interaction network and the disease phenotype network are equal. If *η* > 0.5, then the importance of the gene-gene interaction network is greater than the disease phenotype network. When *η* < 0.5, the gene-gene interaction network is more important than the disease phenotype network is. *P*_0_ and the transition matrix *M* are substituted into Formula (8). After many iterations, steady-state *P*_*∞*_ is denoted as *P*_*∞*_ = [(1 − *η*)*u*_*∞*_ *ηv*_*∞*_]^*T*^. In this way, the steady probabilities *u*_*∞*_ and *v*_*∞*_ are used to rank the genes and disease phenotypes. A web server named GeneWanderer, which is a computational method that prioritizes a set of candidate genes according to their probability to become involved in a particular disease or phenotype using HWRH or diffusion kernel, is used.

### 4.4. Katz

The Katz method is successfully applied to social network link prediction. Predicting the social network link is close to the problem of predicting disease genes. The Katz approach, which is based on a graph, finds the similar nodes for the query nodes in the network [38].

An adjacency matrix *A* is available in an undirected unweighted graph. The Katz approach counts the number of walks of different lengths that connects *i* and *j*. These walks act as the similarity of the two nodes *i* and *j*. (*A* ^*l*^)_*ij*_ is the number of walks of length *l* that connect *i* and *j*. (*A* ^*l*^)_*ij*_ gives a measure of similarity between *i* and *j*. *A* single similarity measure based on the different walk lengths is necessary. The measure is given below, in which *β* is a constant that restrains contributions of longer walks:

(15) $$\begin{array}{c} S_{ij}=\overset{k}{\underset{l=1}{\sum }}\beta_{l}{\left(A^{l}\right)}_{ij}. \end{array}$$

The above measure is denoted as follows:

(16) $$\begin{array}{c} S=\overset{k}{\underset{l=1}{\sum }}\beta_{l}A^{l}. \end{array}$$

If *l* → *∞*, *β*_*l*_ → 0. In this study, setting *β*_*l*_ = *β* ^*l*^ leads to the well-known Katz method:

(17) $$\begin{array}{c} S^{\text{katz}}=\sum_{l\geq 1}\beta^{l}A^{l}={\left(I-\beta A\right)}^{-1}-I, \end{array}$$

where *β* is chosen, such that *β* < 1/||*A*||^2^. In the case of the Katz method, the connections in the graph are weighed so that *A*_*ij*_ is the strength of the connection between nodes *i* and *j*. For the choice of *k*, the sum over infinitely many path lengths is not necessarily considered. According to the experimental results, small values of *k*  (*k* = 3 or *k* = 4) obtain good performance in the task of recommending similar nodes.

The adjacency matrix of the heterogeneous network is denoted as follows:

(18) $$\begin{array}{c} A=\left[\begin{array}{cc} A_{G} & B\\ B^{T} & A_{P} \end{array}\right]. \end{array}$$

One of the advantages of Katz is *A*, which can represent the other species if we want to study human disease phenotypes and other species disease phenotypes.

(19) $$\begin{array}{c} B=\left[\begin{array}{cc} P_{HS} & P_{S} \end{array}\right],\;\;A_{P}=\left[\begin{array}{cc} A_{PHS} & 0\\ 0 & A_{PS} \end{array}\right]. \end{array}$$

Here, *A*_*PHS*_ and *A*_*PS*_ represent human phenotypes and the other species phenotypes, respectively. *P*_*HS*_ and *P*_*S*_ indicate gene-disease phenotype association of human and other species, respectively. When an experiment on human is conducted, set *P*_*S*_ = 0 and *A*_*PS*_ = 0. By synthesizing expressions (18) and (19), we substitute matrix *A* into Formula (17) and obtain the similarity of genes and phenotypes from the similarity matrix.

### 4.5. CATAPULT

Combining data across species by using positive-unlabeled learning techniques is abbreviated to CATAPULT. And CATAPULT is a supervised machine learning method which uses a biased support vector machine (SVM), where the features are derived from walks in a heterogeneous gene-trait network.

Given a query disease phenotype, a gene is not associated with the query phenotype. Scholars report positive association between genes and phenotypes; however, the negative associations are rarely reported. In the CATAPULT approach, the unlabeled gene-disease phenotype pairs act as negative associations. The characteristics of the dataset are that only the positive associations are known, and the negative associations and a large number of unlabeled gene-disease phenotype pairs as negative associations are unknown. The general idea of CATAPULT is that the examples are not known to be negative. The false positives are not penalized heavily, but the false negatives are penalized heavily.

CATAPULT uses a biased SVM to classify the gene-phenotype pairs of humans with a single training phase. This approach draws a random bootstrap sample of a few unlabeled examples from the set of all unlabeled examples and trains a classifier to classify the bootstrap samples as negatives along with the positive samples. CATAPULT also uses the bagging technique to obtain an aggregate classifier by using positive and unlabeled examples. The algorithm description is shown in Algorithm 1. *T* denotes the number of bootstraps, *A* is the set of positive, *n*_+_ denotes the number of examples in *A*, *U* denotes the set of unlabeled gene-disease phenotype pairs, *C*_−_ is a penalty for false positives, and *C*_+_ is a relatively larger penalty for false negatives. The source code can be downloaded from http://marcottelab.org/index.php/Catapult.

Before applying any supervised machine learning approach, extracting the features for gene-disease phenotypes is essential. The features are derived from the paths in the heterogeneous network. For a given gene-disease phenotype pair, different walks of the same length and walks of different lengths can be used as features for the gene-disease phenotype pair.

### 4.6. Meta-Path

The meta-path approach mainly uses the technology of multilabel classification. The multilabel classification method is useful for recognizing disease genes. A gene may exhibit many diseases caused by the gene. In the above example, the gene is an instance, and various diseases are different labels. Given an instance, a large space of all possible label sets may exist, which may be exponential to the number of candidate labels. The frequently used approach to solve the above problem is exploiting correlations among different labels. In the network, exploiting the correlations among different labels denoted by nodes is an advantage.

Meta-path is defined as a sequence of relations in the network. The objects in the network are linked through multiple-type associations. Multiple-type associations help exploit the correlations among different labels for multilabel classification. In recognizing the disease genes, the labels of the genes are diseases, and the labels of the diseases are genes. The explanation of the correlations among genes is summed up in this study.

Given a set of meta-paths among the gene nodes acting as labels, *S*_*l*_ = {*P*_1_, *P*_2_,…, *P*_*cl*_}, the meta-path-based label correlations can be used as follows:

(20) $$\begin{array}{c} \forall i,\;P\left(Y_{i}\;|\;x_{i}\right)=\overset{q}{\underset{k=1}{\prod }}P\left(Y_{i}^{k}\;|\;x_{i},Y_{i}^{P_{1}(k)},Y_{i}^{P_{2}(k)},\ldots ,Y_{i}^{P_{cl}(k)}\right), \end{array}$$

where *P*_*j*_(*k*) denotes the index set of the genes linked to the *k*th gene through the meta-path *P*_*j*_ ∈ *S*_*l*_. *x*_*i*_ denotes the feature vector of node *i* in the input space. *Y*_*i*_ denotes the association between a gene and a gene set. The set of all candidate genes is denoted as *V*_*l*_ = {*l*_1_, *l*_2_,…, *l*_*q*_}, and *Y*_*i*_ is denoted as *Y*_*i*_ = (*Y*_*i*_^1^,*Y*_*i*_^2^,…,*Y*_*i*_^*q*^)^*T*^ ∈ {0,1}^*q*^. In the same way, given a set of meta-paths among disease phenotype nodes acting as instances, *S*_*I*_ = {*P*_1_′,…, *P*_*cI*_′}, the meta-path-based label correlations can be used as follows:

(21) $$\begin{array}{c} P\left(Y\;|\;X\right)\approx \underset{i}{\prod }P\left(Y_{i}\;|\;x_{i},Y_{p_{1}^{\prime }(i)},\ldots ,Y_{p_{cI}^{\prime }(i)}\right), \end{array}$$

where *P*_*j*_′(*i*) denotes the index set of disease phenotypes linked to the *i*th disease phenotype through meta-path *P*_*j*_′ ∈ *S*_*I*_.

To perform multilabel collective classification more effectively in heterogeneous information network, both meta-path-based label correlations and meta-path-based instance correlations are performed simultaneously:

(22) $$\begin{array}{c} P\left(Y\;|\;X\right)\approx \underset{i}{\prod }\;\overset{q}{\underset{k=1}{\prod }}P\left(Y_{i}^{k}\;|\;x_{i},Y_{i}^{p_{j}(k)},\ldots ,Y_{p_{j}^{\prime }\left(i\right)}^{k}\right), \end{array}$$

where the gene is set as the label set, and the disease phenotype is set as the instance set. On the contrary, the disease phenotype is set as the label set, and the gene set is as the instance set.

Some research has proposed algorithms based on multilabel collective classification. We briefly introduce the multilabel collective classification algorithm called PIPL. The algorithm roughly includes the following steps.

1. Meta-path constructions: extracting all nonredundant meta-paths for label correlations and instance correlations.
2. Training initialization: construction of *q* extended training sets for all 1 ≤ *k* ≤ *q*, *D*_*k*_ = {(*x*_*i*_^*k*^, *y*_*i*_^*k*^)} by converting each instance *x*_*i*_ to *x*_*i*_^*k*^ by using the functions in Algorithm 2. Training one classifier on each label by using the extended training sets.
3. Iterative inference: the inference step is an iterative classification algorithm. It updates the testing instance label set predictions and the relational features of label and instance correlations.

## 5. Evaluation Methods

A comprehensive comparison should be conducted among these methods. In the next several paragraphs, we will introduce some of the key comparisons for recognizing the disease genes reported so far.

Cross-validation is the most frequently used approach in evaluating these methods. However, this method is similar to that used in a previous work, which performs leave-one-out. Each of the known gene-disease phenotype associations is taken as a test case, and a set of genes is assigned as the negative control for each test case. In each round of cross-validation, the disease phenotype is held out, a link between the disease phenotype and one of the associated genes is removed, and the link removed gene is added into the test genes. The rank of the test genes is obtained according to the recognizing methods. Several processing approaches are available for the rank, such as the enrichment score, setting a threshold, precision, recall, and receiver-operating characteristic (ROC).

### 5.1. Enrichment Score

Suppose the number of test genes is 100. If a recognition disease gene method ranks the actual disease gene as the highest and is sequenced first, then an enrichment of 50-fold exists. The formula of the enrichment score is Enrichment = 50/rank.

### 5.2. ROC Analysis

ROC analysis denotes the true-positive rate (TPR) versus the false-positive rate (FPR) subject to the threshold dividing the prediction classes. The TPR/FPR is the rate of correctly/incorrectly classified samples of all samples classified to the positive class. To evaluate the scores of disease gene predictions, ROC is explained as a plot of the number of the disease genes above the threshold versus the number of the disease genes below the threshold. The area under the ROC curve for each curve is calculated to compare the different curves obtained by the ROC analysis.

### 5.3. Setting a Threshold

Concordance score is calculated for each test gene. If the true disease gene ranks first based on the concordance score, then the prediction is successful, and precision is used as the proportion of the successful predictions among all predictions. Another evaluation approach is setting a threshold, in which the highest score of all test genes in this case is not less than the threshold. Thus, *recall* is the fraction of true disease genes predicted among all disease genes.

## 6. Materials and Results

In the above section, several recognition disease gene methods and evaluation methods have been mentioned. This part introduces the data used and the comparison results.

**Figure 2:**
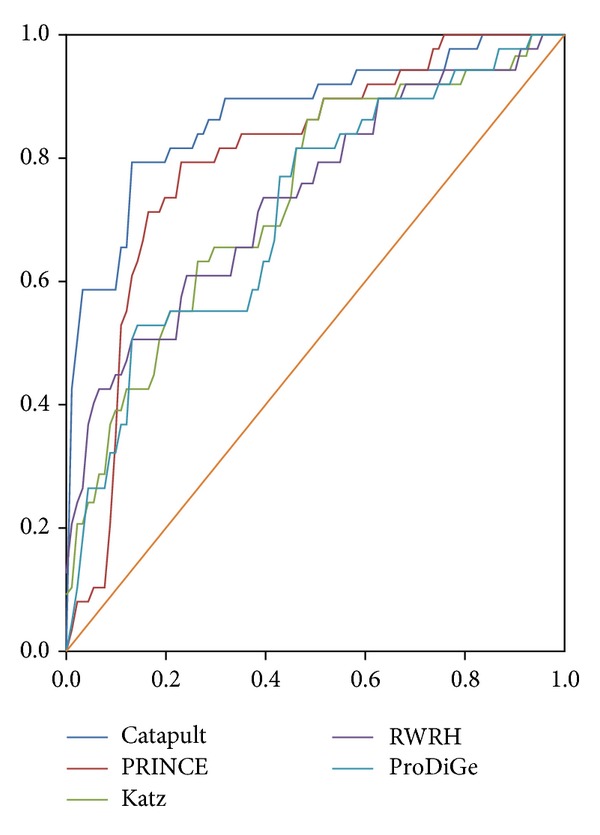
Setting a threshold to compare recognition disease gene methods.

Figure 2 [17] denotes the comparison of different recognition methods by setting a threshold. In Figure 2, six recognizing methods are shown, including Catapult, Katz, ProDiGe, RWRH, PRINCE, and Degree. HumanNet gene network, which is a part of the OMIM dataset, is the dataset used to compare the different recognition methods. Figure 2 shows that Katz and Catapult do better than the others with the HumanNet gene network and the evaluation method.

**Figure 3:**
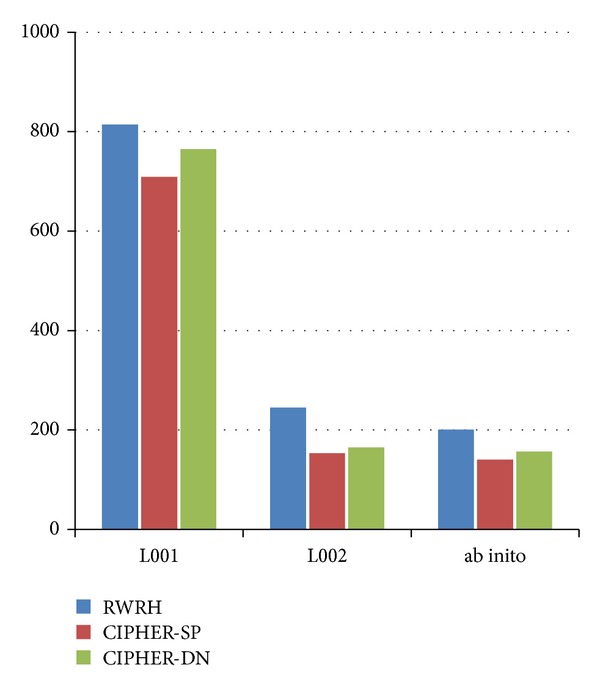
The comparison of different methods.

**Figure 4:**
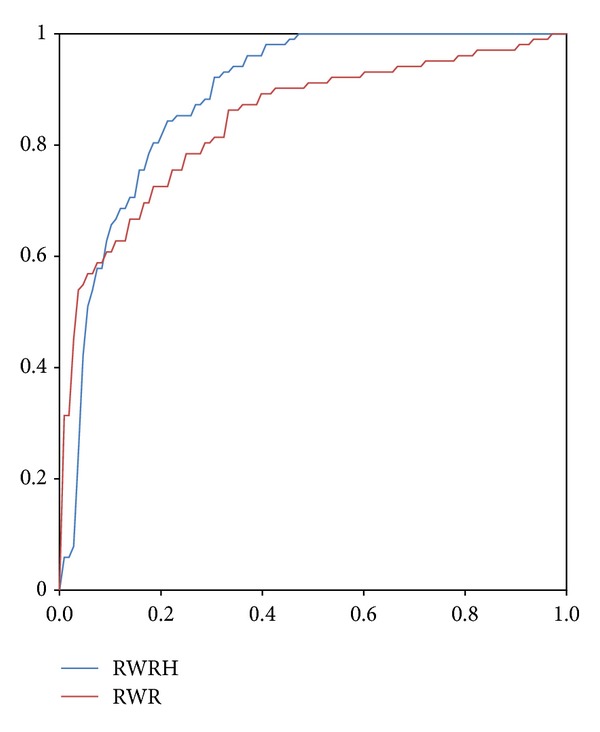
ROC curve of RWR and RWRH.

RWRH is compared with CIPHER-DN and CIPHER-SP. The evaluation experiment is based on gene network containing 34,364 interactions between 8919 genes, the phenotypic similarity matrix between 5080 phenotype entities calculated by using MimMiner, and 1428 gene-phenotype links between 937 genes and 1216 phenotype entities. The comparison result is denoted by Figure 3 When *γ* = 0.7, *λ* = *η* = 0.5, RWRH successfully ranks 814 known disease genes as top 1. The result is denoted by L001 in Figure 3. The column of L002 is the result of removing a known gene-phenotype link and using the phenotype and the rest of the disease genes associated with this phenotype as seed nodes. The identification of disease genes for phenotype from the genome is called *ab initio* prediction. The *ab initio* method removes all the links from a phenotype to disease genes and uses the phenotype entity as seed node to run RWRH. If one of the disease genes associated to the phenotype ranks top 1, then the prediction is successful. The result of *ab initio* is shown in Figure 3. From the L001, L002, and *ab initio*, RWRH is better than CIPHER-SP and CIPHER-DN.  Figure 4 denotes the result of the comparison between RWR and RWRH. Leave-one-out cross-validation is conducted for each disorder. In each cross-validation, a disease gene is selected, and the links between the phenotype entries and the disease gene are removed. The rest of the disease genes and the phenotype entry are used as seed nodes. The selected disease gene and all disease genes in the artificial linkage are ranked by RWRH and RWR. ROC analysis is used to evaluate the two recognizing approaches.

## 7. Conclusion

Identifying disease genes is one of the fundaments of medical care and has been a goal in biology. Although traditional linkage analysis and modern high-throughput techniques often provide hundreds of disease gene candidates, identifying disease genes in the candidate genes by using the biological experiment method time-consuming and expensive. To deal with the above issues, the methods based on networks have been proposed. Many methods based on network have been created to recognize disease genes. In this paper, five typical algorithms based on networks, namely, CIPHER, PRINCE, RWRH, Katz, and CATAPULT, are introduced in detail.

Some novel methods have been put forward to recognize and prioritize disease genes. For instance, BRIDGE [39] takes advantage of multiple regression models with penalty to automatically weight different data sources. A researcher employed the ensemble boosting learning technique to combine variant computational approaches for gene prioritization to improve overall performance [40].

Biological relationships are showed by networks, which brings forth new ideas. A network can be used to denote the association between genes and disease to recognize the gene-disease phenotype and to obtain a more complete understanding of the biological system. Networks have been successfully used in biology. However, combining experiments with networks results in the challenge of defining node similarities. Different ways to define node similarity may lead to different effects.

With the development of biology and the emergence of a large number of relevant data, disease gene research based on networks constantly matures. New machine learning methods and technologies will be used to predict disease genes. Research on disease gene recognition will achieve new breakthroughs. The disease gene research will open a new era of medical treatment.

## Figures and Tables

**Algorithm 1:**
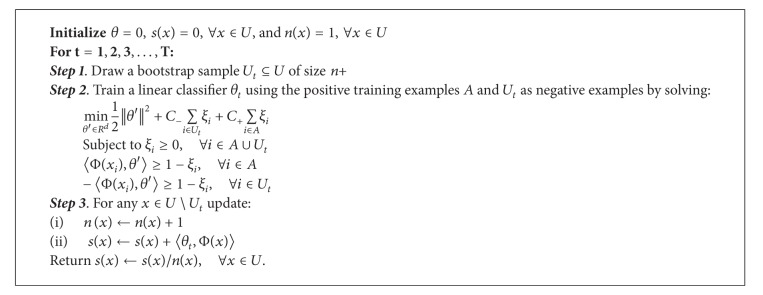
CATAPULT algorithm description.

**Algorithm 2:**
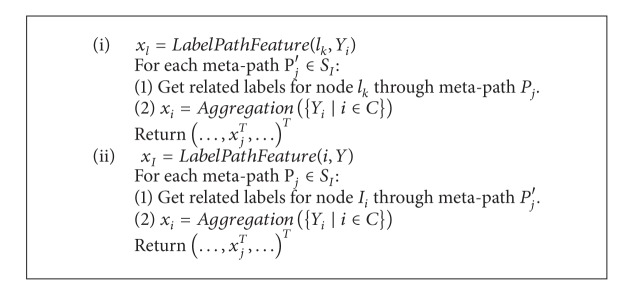
The function of construction relational features for meta-path-based label correlations and meta-path-based instance correlations.
